# WheelSimPhysio-2023 dataset: Physiological and questionnaire-based dataset of immersive multisensory wheelchair simulator from 58 participants

**DOI:** 10.1016/j.dib.2024.110535

**Published:** 2024-05-22

**Authors:** Debora P. Salgado, Sheila Fallon, Yuansong Qiao, Eduardo L. M. Naves

**Affiliations:** a Technological University of the Shannon Midlands Midwest – Athlone Campus, Athlone, Ireland; b Federal University of Uberlandia, Faculty of Electrical Engineering, Uberlandia, Brazil

**Keywords:** HRV, EDA, EEG, Cognitive load, Arousal, XR, Assistive technology

## Abstract

This data paper presents a unique multimodal dataset collected from a comprehensive experiment using a wheelchair training simulator. The dataset consists of quantitative and qualitative data that represents the user's experience and performance. Participants engaged in a series of navigational tasks in a simulated environment under two distinct system configuration conditions: a. a conventional monitor display and b. a virtual reality (VR) headset. The monitor group has a total of 24 participants data while using the simulator with a standard display and then other two groups of 18 and 16 respectively using the VR headset with a different wheelchair's speed profile. It was collected data from total of 58 participants.

The dataset includes physiological data - Heart Rate Variability (HRV), Electrodermal Activity (EDA), Acceleration (ACC), Skin Temperature, Heart Rate (HR), and Blood Volume Pulse (BVP) - collected during both experiments. Additionally, for the standard display condition, more detailed data comprising Electroencephalography (EEG) and eye-tracking metrics were recorded to provide insights into cognitive load and visual attention patterns.

System metrics captured from the simulator provide an objective performance report, including task completion times, error rates (collision of the virtual wheelchair), number of joystick commands. Also, the navigation efficiency data is complemented by post-experiment questionnaires, which gathered subjective responses on user experience, perceived difficulty, the user immersive levels, arousal, and simulator sickness symptoms.

This dataset is valuable for researchers and practitioners in the fields of assistive technology, human-computer interaction, and rehabilitation. It offers metrics to a comprehensive view of how different display technologies influence the user experience in wheelchair simulation training. The data allows for in-depth analysis of physiological responses, cognitive engagement, and subjective perceptions, providing a foundation for future research on effective wheelchair training methodologies and the potential benefits of VR in rehabilitation settings.

Specifications Table

**Table:** 

Subject	Human–Computer Interaction Neuroscience: Cognitive
Specific subject area	User's Quality of Experience Evaluation, Virtual Reality for wheelchair training, physiological data (HRV, EDA, Eye-tracking, EEG, Head-pose), system report (number of commands, collisions and time completions, wheelchair positions, and head movements), post-Experience Assessments (immersion, usability, emotion, cognitive task load, simulator sickness).
Type of data	Raw Data: ­ Physiological data: .csv and .xdf ­ System data: .txt or .csv ­ Questionnaire data: .csv ­ Supplementary data: .pdf and .xlsx
Data collection	- **WheelSimPhysio-2023 dataset** were collected from two types of experiments with the same wheelchair training simulator presented at [1]: ○ **Experiment 1**: the simulator was presented in a PC with a conventional monitor. It was collected data from non-wheelchair users (N_monitor_ =24 participants). We collected a baseline data (5 minutes resting phase), test data (simulation activity) and post-experience assessment (participants answered questionnaires on a paper-based version). ○ **Experiment 2**: the simulator was configured as a fully immersive by using VR HMD, with the same PC and same data collection protocol. It was collected data from 34 participants, split into two groups (N_high-jerk_=18 and N_low-jerk_=16). The first VR group experienced the virtual wheelchair with a high jolt (jerk) sensation when wheelchair's start and stop and the second group had the settings with less jolt sensation.
	
	
	- Hardware/Software specifications: ○ **Wheelchair Simulator**: the application was developed using the Unity game engine in PC machine that operates with monitor or VR HMD. ○ **Physiological data**: the physiological response was collected by using the Empatica E4 wristband. It has four sensors to determine the blood volume pressure (BVP) at a sample rate of 64 Hz, interbeat interval (IBI), Heart Rate (HR) in a sample rate of 1 Hz, electrodermal activity (GSR/EDA) at a sample rate of 4 Hz, XYZ raw acceleration at a sample rate of 32 Hz and the skin temperature at a sample rate of 4 Hz. The EEG data was collected using Mindwave Mobile from Neurosky, is one channel EEG device, the electrodes are dry type, its bandwidth is between 3 and 100 Hz (Hz), with 12 bits of resolution, sample rate is 512 Hz and the transmission is made via Bluetooth. Eye-gaze and head pose were collected using a HP w200 with OpenFace framework. ○ **Supplementary data:** These data consist of templates used for the consent form and questionnaires in .pdf format; the response letters from the institutional ethics committee; and the summary answers (anonymized) from the participants in .xlsx format."
Data source location	Institution: Technological University of The Shannon (Previously named Athlone Technology Institution) City/Town/Region: Athlone, Co. Westmeath Country: Ireland
Data accessibility	Repository name: WheelSimPhysio-2023 dataset [2] Data identification number: doi: 10.17632/z6dfjh596r.2 Direct URL to data: https://data.mendeley.com/datasets/z6dfjh596r/2 Additional information related to do basic pre-processing analysis in the data can be found at the GitHub repository: https://github.com/deborasal/WheelSimPhysio-2023
Related research article	[1] Débora Pereira Salgado, Ronan Flynn, Eduardo Lázaro Martins Naves, and Niall Murray. 2022. A questionnaire-based and physiology-inspired quality of experience evaluation of an immersive multisensory wheelchair simulator. In Proceedings of the 13th ACM Multimedia Systems Conference (MMSys '22). Association for Computing Machinery, New York, NY, USA, 1–11. https://doi.org/10.1145/3524273.3528175

## 1 Value of the Data


 

• *Comprehensive Understanding of User Experience/ User Profiling:* The data is of interest to researchers who are conduction research combing VR with psycho-physiological data. The dataset provides a holistic view of how users interact with and respond to the VR environment, integrating objective performance metrics with subjective perceptions and physiological responses. This comprehensive approach offers deeper insights than any single data type could provide on its own.
• *Contribution to Multidisciplinary Research:* The dataset is valuable for researchers across various fields, including psychology, human-computer interaction, ergonomics, and neuroscience. It offers a rich source of information for studies on human behavior, cognition, emotional response, and physiological reactions in simulated environments.
• *Insights for Broader Applications:* the multimodal dataset provides a rich source for various types of analysis which can contribute to broader fields such as assistive technology, ergonomics, user-centered design for dynamic virtual simulations where the users move in the virtual world but not in the real world.
• *Exploratory Data Analysis:* the data can be used for descriptive, data visualization, time-series and frequency analysis, correlation, regressions, factor analysis and principal component analysis (PCA), Machine Learning and Predictive Modeling, user performance, user experience and comparative analysis.

## 2 Background

In compiling this dataset, our primary motivation was to explore the effectiveness and user response to different display modalities in wheelchair training simulators, a tool increasingly used in rehabilitation and assistive technology. The theoretical backdrop of this study is grounded in the principles of human-computer interaction and the psychology of learning and adaptation in simulated environments [3]. Wheelchair training simulators offer a safe, controlled setting for skill development, but the impact of display technology on user experience and learning outcomes is not thoroughly understood.

By comparing traditional monitor displays with immersive VR headsets, we sought to generate data that could elucidate differences in physiological responses, cognitive load, and user engagement between these modalities. The inclusion of physiological measures (HRV, EDA, ACC, Temperature, HR, BVP), EEG, and eye-tracking in the first experiment, and system performance metrics and post-experience questionnaires in both, provides a comprehensive, multimodal view of the user experience [4].

This dataset complements an original research article that focused on the behavioral outcomes of these modalities in wheelchair training. The data article extends the value of our research by offering a granular view of the physiological and cognitive underpinnings of these outcomes, thereby providing a resource for further research in this evolving field.

## 3 Data Description

**Fig. 1:**
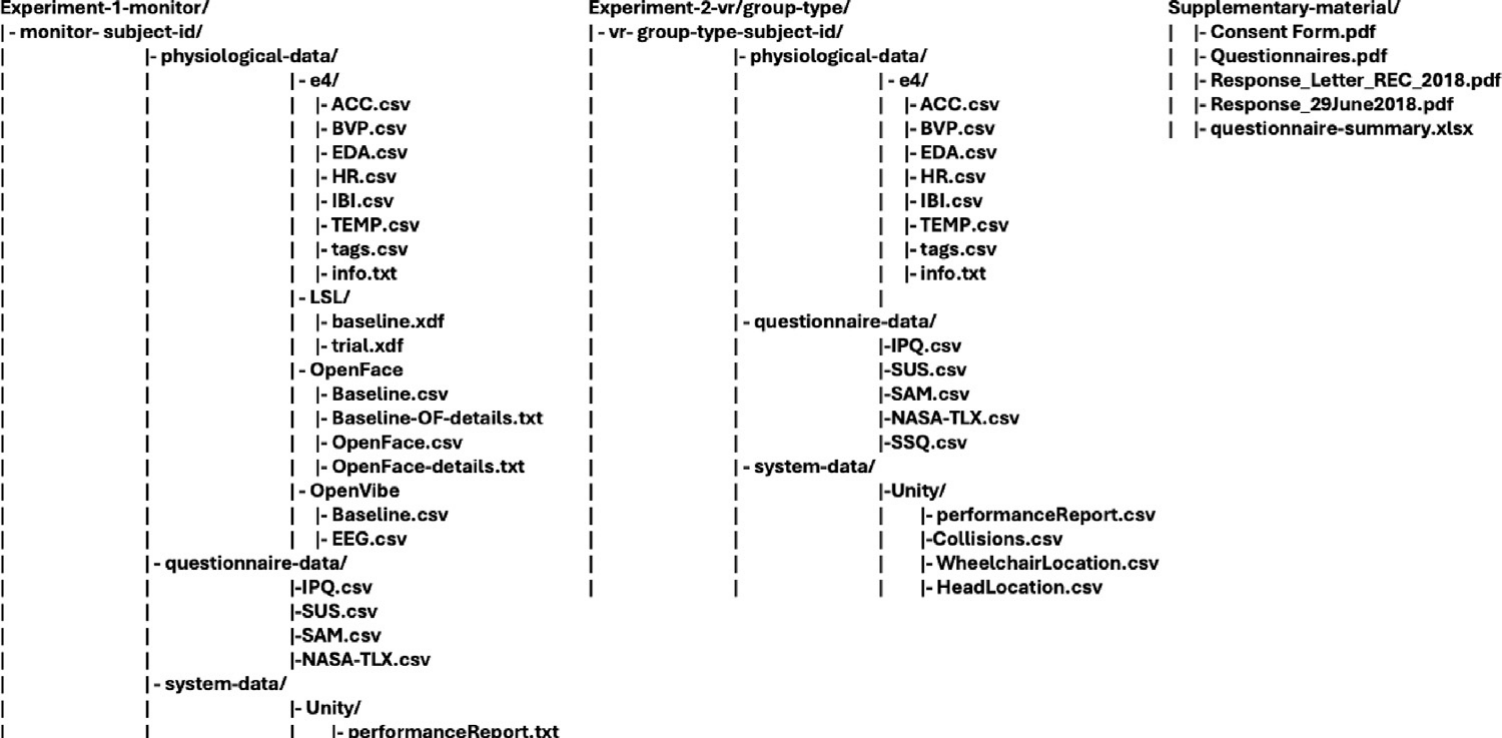
Dataset folder structure.

The presented dataset is in a raw format with supplementary material and annotation that ensures no details are lost and allows reusability of it. The structured of the dataset is presented in Fig. 1. The files are divided per type of experiment, subject and origin of the data folders (physiological, system and questionnaire). The supplementary material folder contains the consent form template, questionnaires template, the responses’ letters from research ethics committee and summary answers from the questionnaire in .xlsx format and anonymized.

The physiological data were collected using Empatica E4 [5]wristband and EEG was using collecting Mindwave [6]. The system data were acquired through the simulator application designed in Unity engine at machine. The questionnaire data were collected as paper based after the experience and were transcribed the results with no transformation in .csv format. The copy of the questionnaires can be found at the supplementary-data folder. Table 1 describes each type of physiological data present in the data.

**Table 1:** Physiological data description.

Folder	File	Description
E4	ACC.csv	Accelerometer data from 3-axis accelerometer sensor in the range [2 g 2 g]. (sampled at 32 Hz) more details of the output format can be found at https://support.empatica.com/hc/en-us/sections/200582445-E4-wristband-data [5]
	BVP.csv	blood volume pressure data from photoplethysmography (PPG). (sampled at 64 Hz)
	EDA.csv	data from the electrodermal activity sensor in μS. (sampled at 4 Hz)
	HR.csv	This file contains the average heart rate values, computed in spans of 10 seconds.
	IBI.csv	inter beat intervals. (intermittent output with 1/64 s resolution)
	TEMP.csv	Data from temperature sensor expressed in degrees on the Celsius (°C) scale (sampled at 4 Hz)
	Tags.csv	file contains a record of the time of each of the event mark times during a session. This event markers has the following tag description: - 1 – Baseline Collection Start - 2 – Baseline Collection Stop - 3 – Experiment Trial Start - 4 – Experiment Trial Stop
	Info.txt	Description of the e4 files

LSL^a^	Baseline.xdf	EEG data during the baseline phase, where the participant is resting for 5 minutes. The file is extensible data forma (XDF) the .XDF files can be parsed by using EEGLAB in matlab or pydxdf in python.
	EEG.xdf	EEG data during the simulation task. The .xdf files also includes the event markers:The .XDF file also included the event markers from the simulation such as: - 1081 – Eyeblink event - 32,769 – Experiment start - 32,773 - Trial start - 898 – Collisions event - 32,774 - Trial Stop - 32,770– Experiment stop

OpenVibe^a^	Baseline.csv	EEG data during the baseline phase that was parsed from .xdf to .csv using OpenVibe platform with LSL as plugin (http://openvibe.inria.fr/how-to-use-labstreaminglayer-in-openvibe/) [12]
	EEG.csv	EEG data during the simulation phase that was parsed from .xdf to .csv using OpenVibe platform with LSL as plugin.

OpenFace^a^	Baseline.csv	Facial landmark, head pose and eye-tracking data during the baseline phase the output format details can be found at the OpenFace wiki page https://github.com/TadasBaltrusaitis/OpenFace/wiki/Output-Format [13]
	Baseline-OF-info.txt	summary information after baseline collection
	OpenFace.csv	Facial landmarks, head pose and eye-tracking data during the trial phase.
	OpenFace-info.txt	Summary information after the trial collection/

a Data available for experiment with monitor display.

Table 2 describes each type of questionnaire data present in the dataset. The participants answered 5 questions from the IGroup Presence Questionnaire (IPQ) [7] to rate their immersion while using the simulator. IPQ is a scale for measuring the sense of presence experienced in a virtual environment. Also, they answered 5 questions from System Usability Scale (SUS) [8] questionnaire for measuring the usability of both simulation setup (Monitor vs. HMD). The cognitive load was evaluated by applying NASA Task Load Index assessment (TLX) [9]. The Self-Assessment Manikin (SAM) [10] was applied to measure the pleasure, arousal and dominance associated with the participant's affective reaction to simulation experienced while performing the predefined task. The Simulator Sickness questionnaire (SSQ) was used for VR HMD experiment to evaluate the level of cybersickness [11].

**Table 2:** Questionnaire data description.

Folder	File	Description
Questionnaire-data	IPQ.csv	5 questions rated from 1–5. (i) general presence “sense of being in the virtual environment (VE)”; (ii) and (iii) spatial presence “the sense of being physically present in VE” (one question is a negative sentence); (iv) involvement “measuring the attention devoted to the experience in VE”; (v) realism “measuring the subjective experience of realism in VE”;
	SUS.csv	5 questions rated from 1–5. (i)and (ii) questions about the participants' opinion how easy are to use the simulator (one question is a negative sentence); (iii) one question asking if other people could learn how to use a wheelchair by using the simulator, (iv) about if the system is inconsistent and (v) if the simulator can be used without any previous knowledge.
	SAM.csv	Valence, Arousal and Dominance scales rated from 1–10
	NASA-TLX.csv	Six subjective subscales as mental, physical, temporal, performance, effort, and frustration demand
	SSQ.csv^a^	The user's score 16 symptoms on a four-point scale (0–3). These scores are grouped into 3 main symptoms (nausea, oculomotor and disorientation) and into a total score.

a Data available for experiment with VR headset.

Table 3 provides an overview of the system data types. It was recorded the commands, collisions, and time for completion of task in the virtual reality application. Also, it was recorded the location of the virtual wheelchair and head movements during the experiments.

**Table 3:** System data description.

Folder	File	Description
System-data	performanceReport.txt	Time series data with the commands, collisions events and final total time, commands and collisions at the end of the report
	performanceReport.csv	.CSV format of performanceReport.txt
	Collisions.csv^a^	Time series data with wheelchair location when a collision happened.
	WheelchairLocations.csv^a^	Time series data of wheelchair locations (path) as coordinates (x,y,z) and rotations (Euler and quaternion system coordinates) in the virtual environment
	HeadLocations.csv^a^	Time series data of head tracking coordinates (x,yz) and rotations (euler and quaternion system coordinates) in the virtual environment.

a Data available for experiment with VR headset.

## 4 Experimental Design, Materials and Methods

### 4.1 Participants

Before the experiments could be undertaken on human test subjects, ethical approval was obtained from the institute's ethics committee. A convenience sampling approach was used to recruit 62 subjects. However, we have selected 58 participants; 4 participants were deemed ineligible for testing via our screening and data collection protocol. Participants were randomly assigned into three groups: monitor/desktop (12 male and 12 females), VR high-jerk sensation (10 male and 8 female) and VR with low jerk sensation (8 males and 8 females). Participants who needed glasses were allowed to wear them during the experiments. It was performed a power analysis by using G*Power 3.1 tool. The power analysis made it possible to identify the appropriate sample size per group; the optimal number was 26. However, with a maximum sample size of 24 and a minimum of 16 were possible to find statistics differences (α <0.05), with a large effect size (d > 0.8), and a power of 80% (1 -β err prob = 0.8).

### 4.2 Wheelchair training simulator setup

**Fig. 2:**
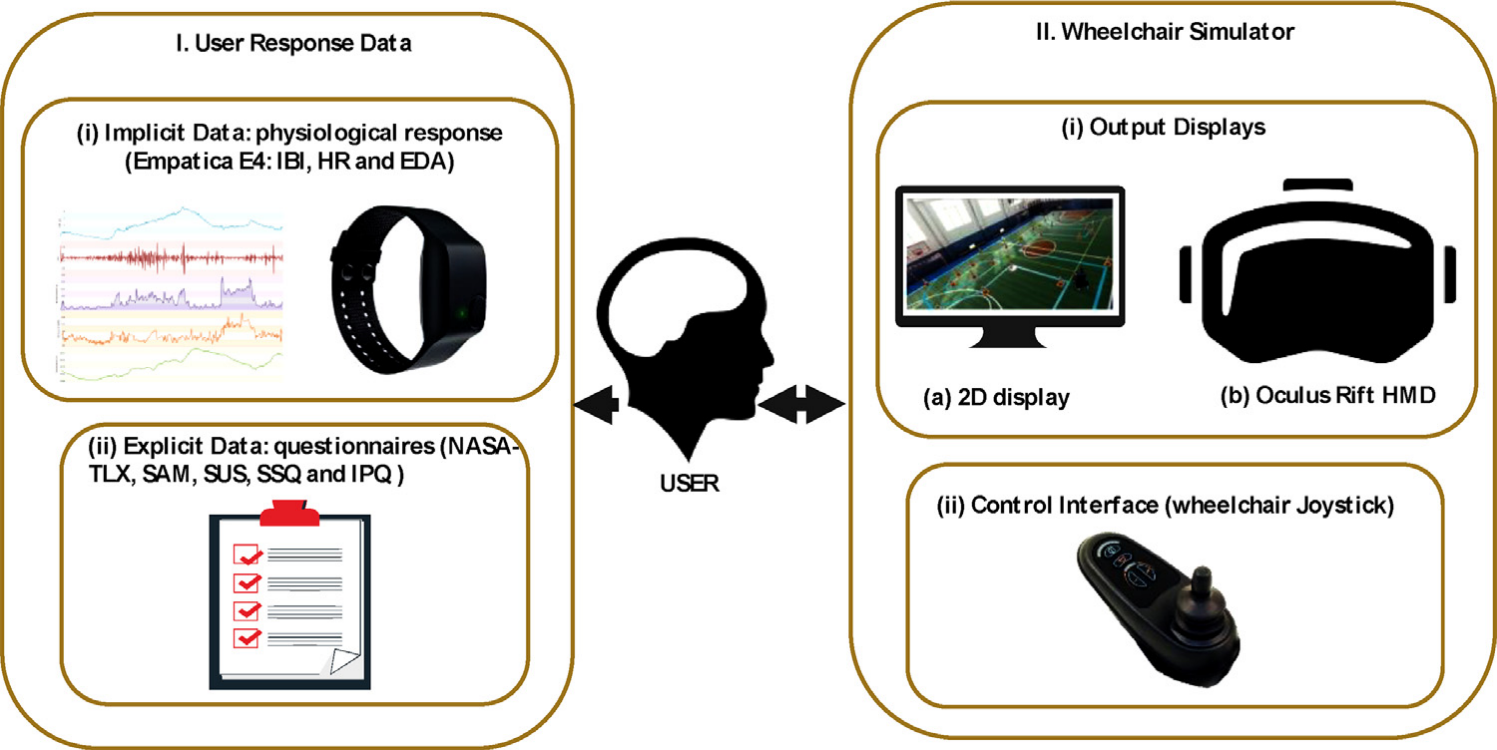
System design.

The virtual environment used in this research was developed using the Unity 3D game engine, version 2017.2.0f3 (64-bit). The PC used was Windows 10 Enterprise which had an Intel CoreTM i7-8700 CPU @3.20 GHz, 16GB RAM with an 8GB NVIDIA GeForce GTX 1080 Graphics Card. The simulation was developed as a training tool, providing inexperience users of electric wheelchairs a method of learning the necessary driving skills without putting themselves at risk. The simulator was operated with Oculus Rift Development kit 2 (DK2) and a with a conventional monitor display. The control interface was using a joystick from a real wheelchair (VR2 model) adapted to be interfaced with PC machine via USB adapter. Fig. 2 represent the components used for data collection with wheelchair training simulation application.

### 4.3 Data synchronization framework

**Fig. 3:**
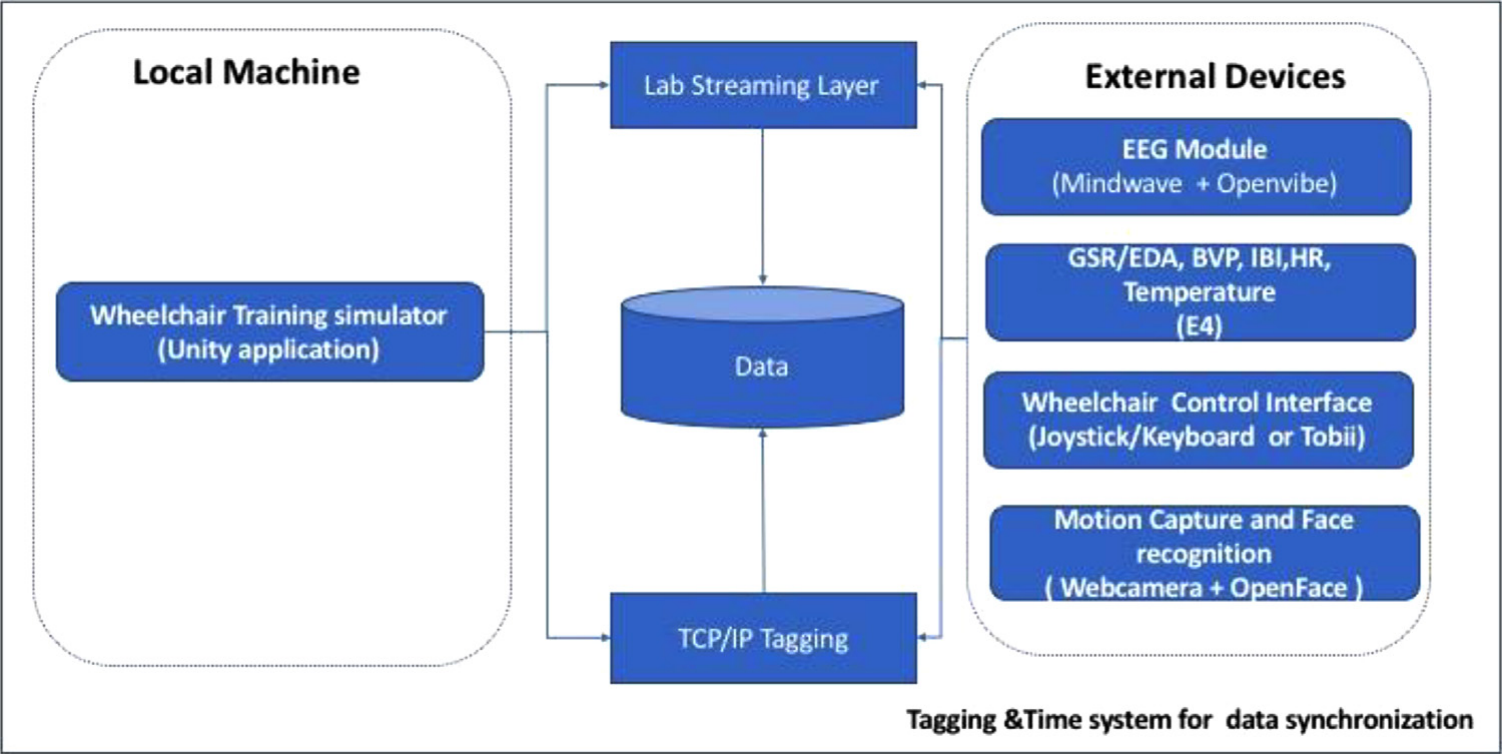
Syncrhonization protocol framework.

The physiological data and simulator data were synchronized in two different approaches is shown on the Diagram in Fig. 3. The first approach is using Lab Streaming Layer (LSL), (https://labstreaminglayer.readthedocs.io/index.html) [14], which is an opensource software for data acquisition, time-synchronization, and real-time access. LSL sends multiple sensor data streams to applications, record and manipulate the data in MATLAB. For our setup, the subject, who was wearing the EEG, is seated in front of a computer running the Unity simulator and drives a wheelchair using a Joystick. Performance metrics and Joystick events from Unity are recorded simultaneously with EEG data from the headset by LSL. The saved data can be post processed and analysed.

Second approach is the TCP/IP tagging, the simulator talks to the external modules (e.g EEG headset and joystick) by socket client-server communication using TCP/IP protocol, with this mechanism was possible to send event markers. The simulator sends the following events: (a) Experiment Start; (b) Experiment Stop; (c) Trial Start; (d) Trial Stop; (e) Baseline Start; (f) Baseline Stop; (g) Button pressed; (h) Button released; (i) Collision (User mistake). The EEG device was used with OpenVibe platform that allows the tagging mechanism from external applications using the socket server and LSL (https://github.com/labstreaminglayer/LSL4Unity) [15]. The E4 was synchronized by using the button event marking and setting the E4’s internal clock with the local machine(https://support.empatica.com/hc/en-us/articles/205672769-Synchronize-your-E4-wristband-for-the-first-time) [16]. In simple words, we have used the LSL synchronization protocol to stamp participants EEG signals with the other modules of system. The same way, the TCP/IP Tagging method validates that the events are synchronized and follows the experimental protocol markers.

### 4.4 Data collection protocol

**Fig. 4:**
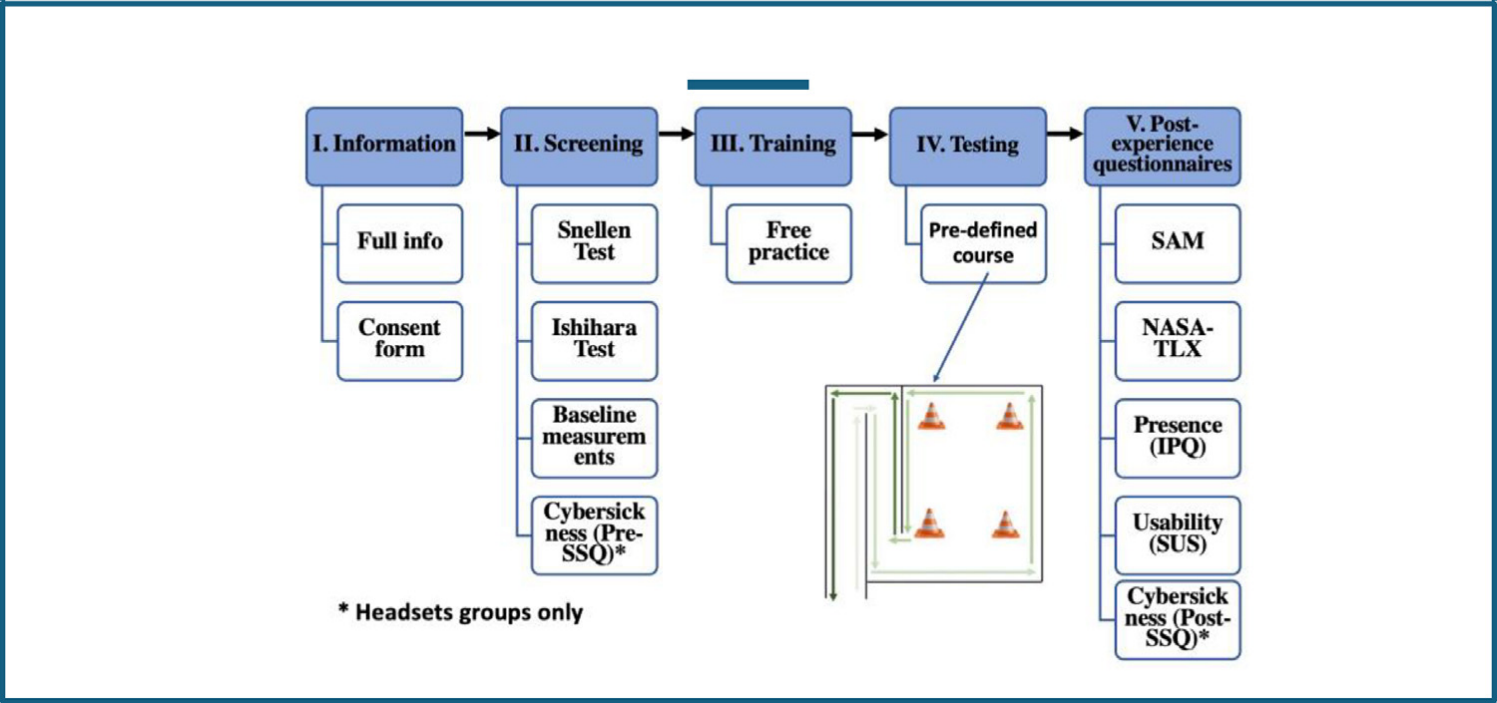
Assessment protocol.

**Fig. 5:**
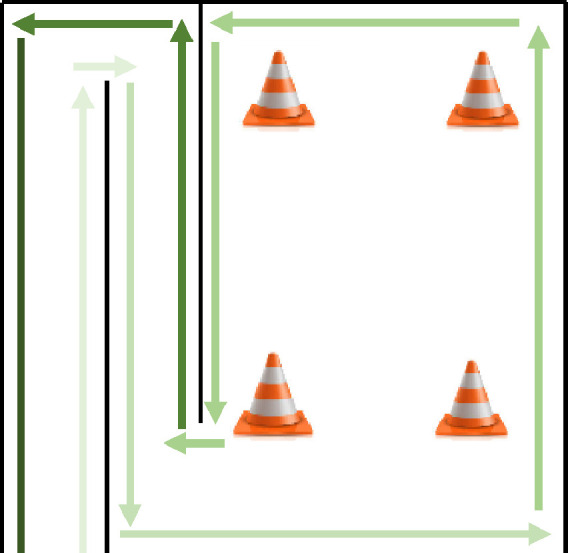
Assessment's Task - Ramp route.

**Fig. 6:**
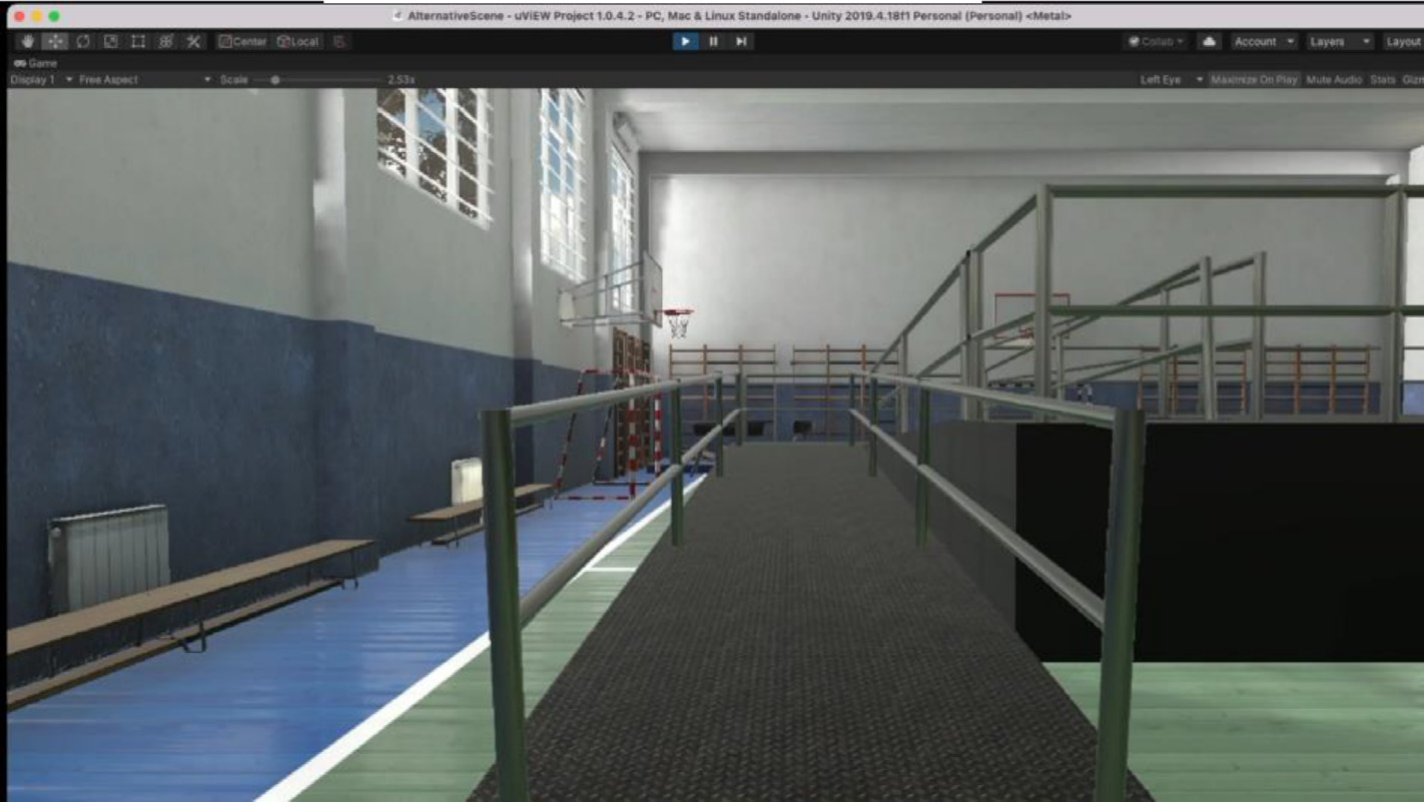
Ramp route view.
v. Post-experience questionnaire: the participants completed the IPQ, SUS, SAM and NASA-TLX questionnaires and SSQ were applied (for the experiment with the VR).

The assessment protocol was categorized into five key phases as per Fig. 4: (I) pre-screening (information phase); (II)screening phase; (III) training phase; (IV) testing phase; and (V) post-experience questionnaire phase. The participants completed the test between 40 and 45 min. Typically, this included a 10-min informative phase, a 10-min screening process, a 5-min training phase, 15-min testing phase and 10-min post-experience questionnaire phase.

i. Information phase: during the pre-screening, each participant was greeted and thanked for their participation. They were provided with a briefing session (virtual or face-to-face) about the study, with an information sheet that described the experiment in full and they will request to sign the consent form.
ii. Screening phase: They were asked about their current state, e.g., if they lack sleep (if they slept less than six hours in the last 24 h), were suspected of being pregnant, or had consumed alcohol in the last 24 h. Upon completion, baseline metrics were captured over a five-minute period using the wearable sensors.
iii. The training phase: each participant experimented with the virtual wheelchair with a joystick in a free practice environment, which is a virtual space without obstacles. The training phase provided opportunity for participant to understand how to control the virtual wheelchair with the control interface (joystick). These lasted approximately 5 min. The training phase content demonstrated how users should interact with the simulator using a combination of gestures within the environment.
iv. Testing phase: the participants were introduced to the tasks as per Figs. 5 and 6. Participants tried the simulator, and the sensing data were captured throughout the assessments.

## Limitations

When discussing the limitations of the data described in the wheelchair training simulator study, several key points need to be considered:

1. Sample Size and Diversity: The representativeness of the data may be limited if the sample size was small or lacked diversity in terms of age and gender and unbalanced groups sizes.
2. Physiological Data Complexity: The interpretation of physiological data such as HRV, EDA, and EEG can be complex and influenced by numerous extraneous factors beyond the scope of the experiment, such as individual differences in stress responses or ambient conditions during testing.
3. VR-Induced Symptoms: Some participants might have experienced discomfort or simulator sickness due to the VR headset, which could have influenced their performance and physiological responses, thereby introducing a confounding variable.
4. Eye-Tracking and EEG Data Limitations: These measures were only collected for the standard display condition. The absence of these data in the VR condition limits the ability to directly compare cognitive load and visual attention across both display modalities.

## Ethics Statement

Consent was obtained in accordance with Declaration of Helsinki and ethical approval was obtained from the Technological University of the Shannon (previously known as Athlone Institute of Technology) Research Ethics committee (REC). The approval letters can be found at the supplementary-folder at [17].

## CRediT authorship contribution statement

**Debora P. Salgado:** Conceptualization, Methodology, Software, Validation, Formal analysis, Investigation, Data curation, Writing – original draft. **Sheila Fallon:** Supervision, Writing – review & editing, Validation. **Yuansong Qiao:** Supervision, Writing – review & editing, Validation. **Eduardo L. M. Naves:** Supervision, Writing – review & editing, Validation, Methodology.

## Data Availability

WheelSimPhysio-2023 (Original data) (Mendeley Data).
